# RNase III coding genes modulate the cross-kingdom biofilm of *Streptococcus mutans* and *Candida albicans*

**DOI:** 10.3389/fmicb.2022.957879

**Published:** 2022-09-30

**Authors:** Yangyu Lu, Lei Lei, Yalan Deng, Hongyu Zhang, Mengying Xia, Xi Wei, Yingming Yang, Tao Hu

**Affiliations:** ^1^State Key Laboratory of Oral Diseases, Department of Preventive Dentistry, National Clinical Research Center for Oral Diseases, West China Hospital of Stomatology, Sichuan University, Chengdu, China; ^2^Guangdong Provincial Key Laboratory of Stomatology, Department of Operative Dentistry and Endodontics, Hospital of Stomatology, Guanghua School of Stomatology, Sun Yat-sen University, Guangzhou, China

**Keywords:** *Streptococcus mutans*, *rnc* gene, *Candida albicans*, *DCR1* gene, cross-kingdom biofilm

## Abstract

*Streptococcus mutans* constantly coexists with *Candida albicans* in plaque biofilms of early childhood caries (ECC). The progression of ECC can be influenced by the interactions between *S. mutans* and *C. albicans* through exopolysaccharides (EPS). Our previous studies have shown that *rnc*, the gene encoding ribonuclease III (RNase III), is implicated in the cariogenicity of *S. mutans* by regulating EPS metabolism. The *DCR1* gene in *C. albicans* encodes the sole functional RNase III and is capable of producing non-coding RNAs. However, whether *rnc* or *DCR1* can regulate the structure or cariogenic virulence of the cross-kingdom biofilm of *S. mutans* and *C. albicans* is not yet well understood. By using gene disruption or overexpression assays, this study aims to investigate the roles of *rnc* and *DCR1* in modulating the biological characteristics of dual-species biofilms of *S. mutans* and *C. albicans* and to reveal the molecular mechanism of regulation. The morphology, biomass, EPS content, and lactic acid production of the dual-species biofilm were assessed. Quantitative real-time polymerase chain reaction (qRT-PCR) and transcriptomic profiling were performed to unravel the alteration of *C. albicans* virulence. We found that both *rnc* and *DCR1* could regulate the biological traits of cross-kingdom biofilms. The *rnc* gene prominently contributed to the formation of dual-species biofilms by positively modulating the extracellular polysaccharide synthesis, leading to increased biomass, biofilm roughness, and acid production. Changes in the microecological system probably impacted the virulence as well as polysaccharide or pyruvate metabolism pathways of *C. albicans*, which facilitated the assembly of a cariogenic cross-kingdom biofilm and the generation of an augmented acidic milieu. These results may provide an avenue for exploring new targets for the effective prevention and treatment of ECC.

## Introduction

Early childhood caries (ECC) is an aggressive form of tooth decay in children younger than 6 years, with a high prevalence of 70% in disadvantaged groups worldwide (James et al., 2018; Peres et al., 2019; Duan et al., 2021). *Streptococcus mutans* (*S. mutans*) is a critical pathogen of dental caries due to its acidogenicity, aciduricity, and capacity to develop cariogenic biofilms by synthesizing exopolysaccharides (EPS) (Lemos et al., 2019). As the main virulence factor of *S. mutans*, EPS are conducive to the energy storage, adhesion, and three-dimensional (3D) architecture of dental plaque biofilms (Koo et al., 2017; Yang et al., 2019). Another microbe frequently detected with *S. mutans* in plaque biofilms of ECC is *Candida albicans* (*C. albicans*), an opportunistic fungal pathogen usually isolated from the human oral cavity (Ponde et al., 2021; Ramadugu et al., 2021). *C. albicans* is closely correlated with the occurrence and development of ECC or persistent apical periodontitis in which the pathogens are difficult to eradicate especially in molars with complicated root canal morphology (Zhang et al., 2011a,b; Mustafa et al., 2022). Children with oral *C. albicans* have a more than 5 times higher possibility of developing ECC than those without *C. albicans* (Xiao et al., 2018b). *C. albicans* can strengthen the ability of microflora to produce or tolerate acid, elevate the abundance of *S. mutans*, and enhance the activity of glucosyltransferases (Gtfs) secreted by *S. mutans* in plaque biofilms of severe early childhood caries (S-ECC) (Xiao et al., 2018a). These findings suggest that the coexistence of *S. mutans* and *C. albicans* is closely related to the progression of ECC. In the co-culture biofilm, the presence of *C. albicans* improves the carbohydrate metabolism of *S. mutans* and affects its environmental fitness and biofilm formation (He et al., 2017). Mannans located in the cell wall of *C. albicans* binds to *S. mutans*-derived GtfB to regulate EPS formation, mechanical stability, and colonization of dual-species biofilms (Hwang et al., 2017; Khoury et al., 2020). *S. mutans*-secreted GtfB, in turn, can not only break sucrose down into monosaccharides that are efficiently utilized by *C. albicans* to enhance its acid production but also improve fungal colonization onto the tooth surface and antifungal resistance to fluconazole (Kim et al., 2018; Ellepola et al., 2019). Transcriptomics and proteomics studies have implied that genes and proteins of *C. albicans* associated with polysaccharide metabolism and cell morphogenesis are significantly upregulated in dual-species biofilms of *S. mutans* and *C. albicans* (Ellepola et al., 2019). Therefore, EPS play important roles in the cross-kingdom interaction between *S. mutans* and *C. albicans*.

Ribonuclease III (RNase III) is the endonuclease in bacteria and eukaryotes that participates in RNA biogenesis to control gene expression (Svensson and Sharma, 2021). Our previous studies have illuminated that the gene encoding *S. mutans* RNase III, *rnc*, enhances the EPS synthesis and cariogenicity of bacterial biofilms through non-coding RNAs that target *vicR*, the gene encoding a response regulator of EPS metabolism (Lei et al., 2018, 2020). On the one hand, *rnc* impairs *vicR* expression by affecting the generation of microRNA-size small RNA 1657 (msRNA 1657), which can bind to the 5′-UTR regions of the *vicR* mRNA (Lei et al., 2019). On the other hand, *rnc* increases the expression of *vicR* antisense (AS*vicR*) RNA, thus influencing the transcriptional level of the *vicR* gene (Lei et al., 2020). *C. albicans* also has *DCR1* as the sole functional RNase III, which is instrumental in small interfering RNA (siRNA), ribosomal RNA (rRNA), and small nuclear RNA (snRNA) generation (Drinnenberg et al., 2009; Bernstein et al., 2012). Notably, although deletions of one copy of the *DCR1* gene in diploids do not restrict *C. albicans* growth, mutants deficient in both copies of the *DCR1* allele cannot be recovered, indicating that *DCR1* is essential for *C. albicans* survival (Bernstein et al., 2012). In summary, as RNase III-coding genes in bacteria and eukaryotes, *rnc* regulates extracellular matrix synthesis and cariogenic characteristics of *S. mutans* biofilms at the post-transcriptional level, while *DCR1* catalyzes the production of non-coding RNAs in *C. albicans*. However, whether *rnc* and *DCR1* affect the EPS and cariogenicity of cross-kingdom biofilms of *S. mutans* and *C. albicans* has not yet been elucidated.

In this study, *C. albicans DCR1* low-expressing and overexpressing strains were constructed and co-cultured with *S. mutans* wild-type or *rnc*-mutant strains to explore the roles of *rnc* and *DCR1* in the modulation of dual-species biofilms of *S. mutans* and *C. albicans*. We demonstrated that *DCR1* regulated the fungal yeast-to-hyphae transition, spatial structure, acid production, and glucan/microorganism ratio of biofilms, while *rnc* prominently contributed to cross-kingdom biofilm formation by boosting extracellular polysaccharide synthesis. Alterations of the microecosystem caused by *rnc* might affect the virulence of *C. albicans* that facilitated the assembly of a cariogenic cross-kingdom biofilm and the formation of an intensive acidic milieu. According to these results, *rnc* and *DCR1* may be new potential targets for ECC prevention and treatment.

## Materials and methods

### Microorganisms and construction of mutant strains

The strains and plasmids used in this study are summarized in Table 1. *C. albicans* SC5314 and CAI4, and *S. mutans* UA159 were kindly provided by the State Key Laboratory of Oral Diseases (Sichuan University, Chengdu, China). *S. mutans* strains were cultured on brain heart infusion (BHI; BD, Franklin Lakes, United States) medium with erythromycin or spectinomycin when necessary at 37°C anaerobically (10% CO_2_, 80% N_2_, 10% H_2_). *C. albicans* SC5314 and CAI4 were grown in the YPD medium (1% yeast extract, 2% peptone, and 2% dextrose) at 30°C with shaking (150 rpm) under aerobic conditions. A *C. albicans DCR1* low-expressing strain (*DCR1/dcr1*Δ) was constructed by deleting one copy of the *DCR1* gene using plasmid p5921 according to the URA-Blaster method (Janik et al., 2019). Upstream and downstream fragments of *DCR1* from the chromosome were inserted into both ends of the *hisG-URA3-hisG* cassette of p5921 to obtain the recombinant plasmid pUC-*DCR1-URA3* to replace one of the *DCR1* alleles in *C. albicans*. The open reading frame (ORF) of the *DCR1* gene was cloned into the pCaEXP plasmid to establish the recombinant vector pCaEXP-*DCR1*, which could integrate into the *RP10* locus of the *C. albicans* genome to create a *DCR1* overexpressing strain (*DCR1^OE^*) (Jia et al., 2011; Basso et al., 2017; Znaidi et al., 2018). All recombinant plasmids were transformed into *C. albicans* CAI4 utilizing the Yeast Transformation Kit (Sigma-Aldrich, St Louis, United States). The mutagenic strains were selected on a synthetic dropout (SD) medium without uridine, methionine, and cysteine and then verified by PCR, DNA sequencing, and quantitative real-time polymerase chain reaction (qRT-PCR) with the primers listed in Supplementary Table 1. The *rnc*-deficient strain (Smurnc) was constructed by an erythromycin cassette based on homologous recombination. Meanwhile, the recombinant plasmid pDL278 containing the *rnc* ORF and promoter region was transformed into *S. mutans* UA159 to generate an *rnc* overexpressing strain (Smurnc^+^), as previously described (Lau et al., 2002; Lei et al., 2020).

**TABLE 1:** Microbial strains and plasmid used in this study.

Strains, plasmid	Relevant characteristics	Source or reference
***S. mutans* strains**		
UA159	Parent strain	ATCC 700610
Smurnc	UA159 *rnc: ermAM*; Erm^r^	Lei et al., 2020
Smurnc^+^	UA159 derived; pDL278-*rnc*; Spec^r^	Lei et al., 2020
***C. albicans* strains**		
SC5314	Parent strain	ATCC MYA-2876
CAI4	*ura3*Δ*:imm434*/*ura3*Δ*:imm434*	Fonzi and Irwin, 1993
*DCR1/dcr1*Δ	*dcr1*Δ*: hisG-URA3-hisG*/*DCR1*	This study
*DCR1* ^OE^	*rp10*Δ*:*pCaEXP-*DCR1*-*URA3*/*RP10*	This study
**Plasmid**		
pDL278	*Escherichia coli*-*Streptococcus* shuttle vector; Spec^r^	Wen et al., 2011
pDL278-*rnc*	Recombinant and expression of *rnc*; Spec^r^	Lei et al., 2020
p5921	Containing *hisG-URA3-hisG*; Amp^r^	Mao et al., 1999
pUC-*DCR1-URA3*	p5921 derived; containing *DCR1* upstream and downstream sequence	This study
pCaEXP	Containing *RP10* sequence; Amp^r^	Care et al., 1999
pCaEXP-*DCR1*	pCaEXP derived; recombinant and expression of *DCR1*	This study

Erm, erythromycin; Spec, spectinomycin; Amp, ampicillin.

### Biofilm formation

*Streptococcus mutans* and *C. albicans* were grown to mid-exponential phase and co-cultured in YNBB medium (0.67% YNB, 75 mM Na_2_HPO_4_-NaH_2_PO_4_, 0.5% sucrose, and essential amino acids) at the concentrations of 2 × 10^6^ colony forming units (CFU)/ml (*S. mutans*) and 2 × 10^4^CFU/ml (*C. albicans*). Cross-kingdom biofilms were formed in microtiter plates at 37°C with 5% CO_2_ for 24 h (Sztajer et al., 2014; Liu et al., 2017).

### Biofilm observation and assessment

The structure of cross-kingdom biofilms was analyzed by scanning electron microscopy (SEM; FEI, Hillsboro, United States). Briefly, after being cultivated on sterile polystyrene slides, the biofilms were washed two times with sterile phosphate-buffered saline (PBS) and fixed with 2.5% glutaraldehyde at 4°C for 4 h in the dark, followed by serial dehydration with ethanol solutions (30, 50, 75, 85, 95, and 99%) and coating with gold for image collection. The biomass of the biofilms was quantified by the crystal violet (CV) microtiter assay. After incubation in 24-well polystyrene plates, the biofilms were stained with 0.1% (w/v) CV for 10 min at room temperature when the planktonic cells and supernatant were gently removed. The specimens were rinsed carefully with water, and then 33% acetic acid was added to dissolve the dye under gentle shaking at 37°C for 5 min. Finally, biofilm formation was detected by the absorbance of acetic acid at 575 nm.

### Lactic acid generation evaluation

The mature biofilms were cultured in buffered peptone water (BPW; Hopebio, Qingdao, China) supplemented with 0.2% (w/v) sucrose at 37°C with 5% CO_2_ for 3 h after rinsing with PBS (Chen et al., 2020). The concentrations of lactic acid produced by the biofilms were calculated based on the standard curve according to the instructions of the Lactic Acid Assay Kit (Nanjing Jiancheng, China).

### Exopolysaccharide distribution in biofilms

Glucan in the biofilms that grew in polystyrene cell culture dishes with modified bottoms was stained with Alexa Fluor 647 (Invitrogen, Waltham, United States) at a final concentration of 1 μl/ml during incubation. Microorganisms were labeled with 2.5 μM SYTO 9 green fluorescent dye (Invitrogen), while *C. albicans* was stained with calcofluor white dye (Sigma-Aldrich) (Punjabi et al., 2020). The distribution of microbes or glucan was detected by confocal laser scanning microscopy (CLSM; Olympus, Tokyo, Japan). Biofilms were reconstructed, and imaging biomass quantification was analyzed using Imaris version 7.2.3 (Bitplane, Zurich, Switzerland) (Lei et al., 2020).

### Exopolysaccharide production measurement

The biofilms were cultivated in 6-well polystyrene plates before the EPS content was determined by the anthrone method. Water-insoluble EPS extraction from dual-species biofilms was performed according to previous protocols with modifications (Lei et al., 2020). Biofilms were gently washed with PBS, collected by scraping, and centrifuged at 4^°^C (2,422 × *g*) for 15 min. The precipitates were solubilized in 1 M NaOH (Sigma, United States) by incubation at 37°C for 2 h at 120 rpm. After centrifugation (2,422 × *g*) at 4°C for 15 min, the supernatant containing water-insoluble EPS was collected and diluted with an anthrone-sulfuric acid reagent to a final concentration of 25% (v/v). The mixed samples were blended and incubated at 95°C for 6 min, and the absorbance was measured at an optical density of 625 nm.

### Characteristics of the biofilm surface

The surface morphology and roughness of the biofilm were visualized and examined by atomic force microscopy (AFM). In brief, the biofilms cultured on polystyrene slides were mildly washed with PBS and dried in the air, followed by the measurement of surface roughness, which is represented by the arithmetic mean deviation of the profile (Ra) counted by an STM9700 system (Shimadzu, Tokyo, Japan) using a cantilever probe (HYDRA-ALL-G-20, AppNANO, Syracuse, United States) in contact mode (Deng et al., 2021). The scanning range was 10 × 10 μm.

### RNA isolation and quantitative real-time polymerase chain reaction assays

Biofilm cells were collected by scraping into PBS and harvested by centrifugation (4,500 × *g*) at 4°C for 10 min. Total RNA was extracted by a MasterPure™ complete DNA and RNA purification kit (Lucigen, Middleton, United States). Purification and reverse transcription of the isolated RNAs were performed by using the RevertAid First Strand cDNA Synthesis Kit (Thermo Scientific, Waltham, United States) with gDNA Eraser (Takara, Dalian, China). qRT-PCR was conducted using TB Green^®^ *Premix Ex Taq*™ (Takara) in a LightCycler 480 System (Roche, Vaud, Switzerland). All primer sequences are listed in Supplementary Table 1. The relative gene expression level of virulence factors of *C. albicans* was normalized to the gene expression level of the reference gene *ACT1*, and the data were interpreted as fold changes based on the control group according to the 2^–ΔΔCt^ method.

### Transcriptome sequencing and data analysis

Total RNA was quantified and qualified using a NanoDrop 2000 instrument (Thermo Scientific) and the 2100 Bioanalyzer (Agilent, Santa Clara, United States), respectively. The mRNA was isolated by the polyA selection method using oligo(dT) magnetic beads, followed by chemical fragmentation, and then double-stranded cDNA was prepared using a SuperScript double-stranded cDNA synthesis kit (Invitrogen) with random hexamer primers (Illumina, San Diego, United States). Libraries were size-selected for end-repaired cDNA target fragments of 300 bp on 2% low-range ultra agarose, followed by PCR amplification. Subsequently, RNA-Seq was conducted with the Illumina HiSeq xten/NovaSeq 6000 sequencer (2 × 150 bp read length). The differential expression genes (DEGs) were classified by Clusters of Orthologous Groups (COG). Furthermore, we performed Gene Ontology (GO) functional enrichment and Kyoto Encyclopedia of Genes and Genomes (KEGG) pathway analysis to identify which DEGs were significantly enriched in GO terms and metabolic pathways using Fisher’s exact tests.

### Statistical analysis

Statistical analysis was performed using SPSS 16.0 (SPSS Inc., Chicago, United States), and differences in data were considered significant if *P* < 0.05. Data normality and the homogeneity of variance were tested by the Shapiro–Wilk method and Bartlett method, respectively. If the data obeyed the homogeneity of variance, one-way analysis of variance (ANOVA) with Fisher’s least significant difference (LSD) multiple-comparison test was used to examine the statistical significance; otherwise, Dunnett’s T3 multiple-comparison test was used.

## Results

### The *DCR1* gene modulated the fungal yeast-to-hyphae transition, spatial structure, acid production, and glucan/microorganism volume ratio of the cross-kingdom biofilm

**FIGURE 1:**
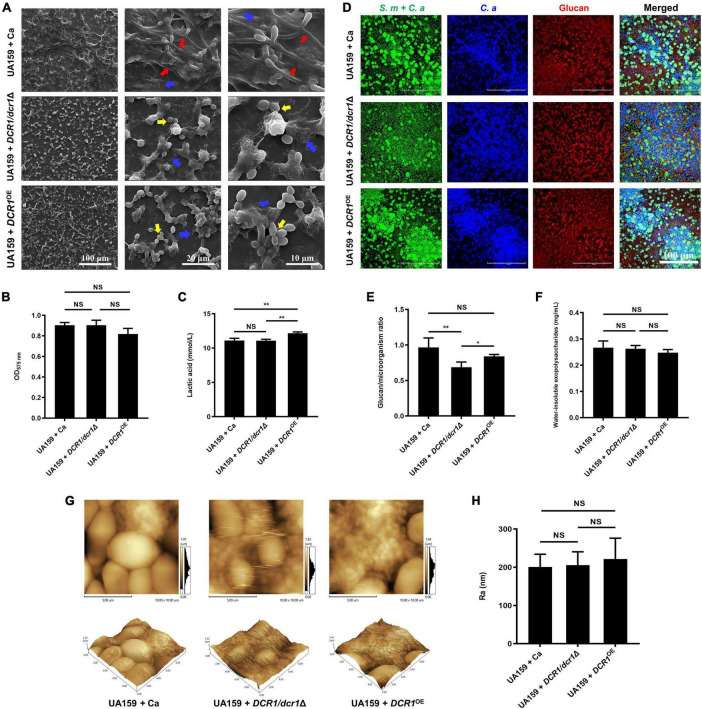
The effects of *DCR1* gene on formation and exopolysaccharide synthesis of cross-kingdom biofilm. **(A)** Biofilms architecture was observed by scanning electron microscopy (SEM). Blue arrows, *S. mutans*; red arrows, *C. albicans* hyphae; yellow arrows, *C. albicans* yeast cells. Images were taken at 1,000 × (scale bar, 100 μm), 5,000 × (scale bar, 20 μm), and 10,000 × (scale bar, 10 μm) magnifications, respectively. **(B)** Biofilms volume was quantified by CV microtiter assay. **(C)** Lactic acid generation was assessed by the Lactic Acid Assay Kit. **(D,E)** Exopolysaccharide production and distribution in biofilms were visualized by confocal laser scanning microscopy (CLSM), and the ratio of glucan to microbes was calculated using Imaris version 7.2.3. Green, total microbes (SYTO 9); blue, *C. albicans* (calcofluor white); red, glucan (Alexa Fluor 647). **(F)** Water-insoluble exopolysaccharides from samples were measured by the anthrone method. **(G,H)** Biofilm morphology was detected by AFM and the surface roughness average (Ra) of biofilms was evaluated. Experiments were performed in biological triplicate. *P < 0.05, **P < 0.01.

We successfully constructed a *DCR1* low-expressing mutant *DCR1/dcr1*Δ and a *DCR1* overexpressing mutant *DCR1*^OE^ (Supplementary Figure 1). The biofilm of UA159 + Ca formed a pronounced 3D structure, and *C. albicans* was present mainly in the hyphal form (Figure 1A). Nevertheless, the cross-kingdom biofilms containing *DCR1/dcr1*Δ or *DCR1*^OE^ seemed to lack skeletal architecture, and there were mostly *C. albicans* yeast cells (Figure 1A), which was in line with the CLSM results (Figure 1D). The glucan/microorganism ratio was used to reflect the capacity of microbial strains to produce glucan. The biofilm of UA159 + *DCR1/dcr1*Δ exhibited a conspicuously decreased glucan/microorganism volume ratio (Figure 1E), while UA159 + *DCR1*^OE^ displayed increased lactic acid production compared to UA159 + Ca (Figure 1C). We also used the Ra (nm) value as an indication of the biofilm surface roughness. There was no significant difference in biomass (Figure 1B), biofilm roughness (Figures 1G,H), or total content of water-insoluble EPS (Figure 1F) among the three groups.

### The *rnc* gene promoted the exopolysaccharide synthesis and cariogenic characteristics of cross-kingdom biofilms

**FIGURE 2:**
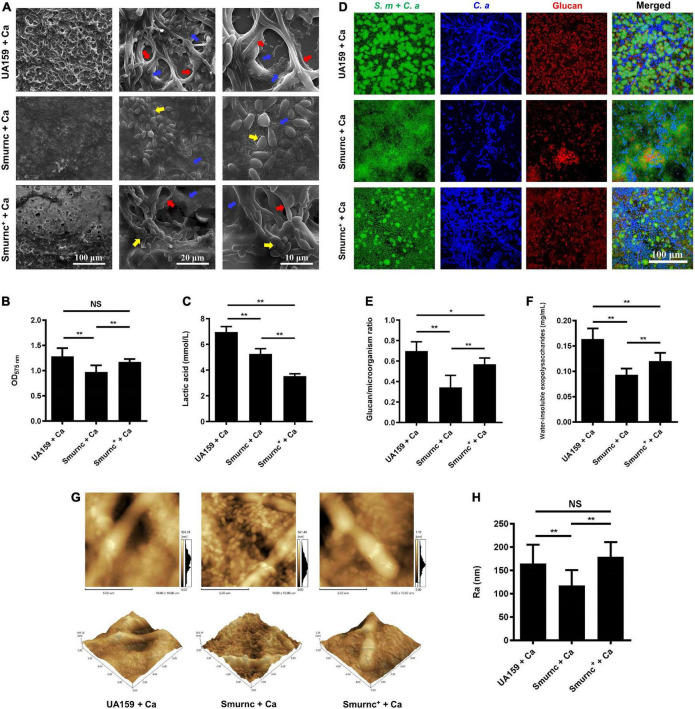
The regulation of *rnc* gene in cariogenic characteristics of cross-kingdom biofilm. **(A)** SEM observation of biofilm architecture. Blue arrows, *S. mutans*; red arrows, *C. albicans* hyphae; yellow arrows, *C. albicans* yeast cells. Images were taken at 1,000 × (scale bar, 100 μm), 5,000 × (scale bar, 20 μm), and 10,000 × (scale bar, 10 μm) magnifications, respectively. **(B)** CV microtiter assay was used to quantify the biofilm biomass. **(C)** Lactic acid generation was assessed by the Lactic Acid Assay Kit. **(D,E)** Exopolysaccharide production and distribution in biofilms were visualized by CLSM. Green, total microbes; blue, *C. albicans*; red, glucan. The glucan to microbes ratio was calculated using Imaris version 7.2.3. **(F)** Water-insoluble exopolysaccharides from biofilms were measured by the anthrone method. **(G,H)** Biofilm morphology was detected by AFM, and the surface roughness indicated by Ra (nm) was evaluated. Experiments were performed in biological triplicate. *P < 0.05, **P < 0.01.

**FIGURE 3:**
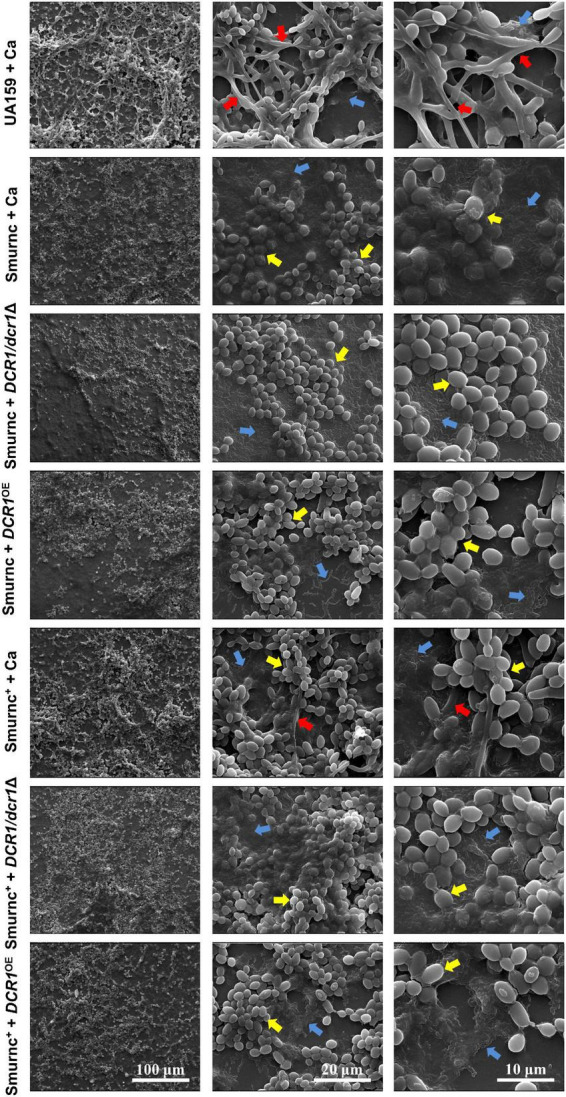
The effects of genes coding RNase III on the architecture of cross-kingdom biofilms were observed by SEM. Blue arrows, *S. mutans*; red arrows, *C. albicans* hyphae; yellow arrows, *C. albicans* yeast cells. All images were taken at 1,000 × (scale bar, 100 μm), 5,000 × (scale bar, 20 μm), and 10,000 × (scale bar, 10 μm) magnifications, respectively.

**FIGURE 4:**
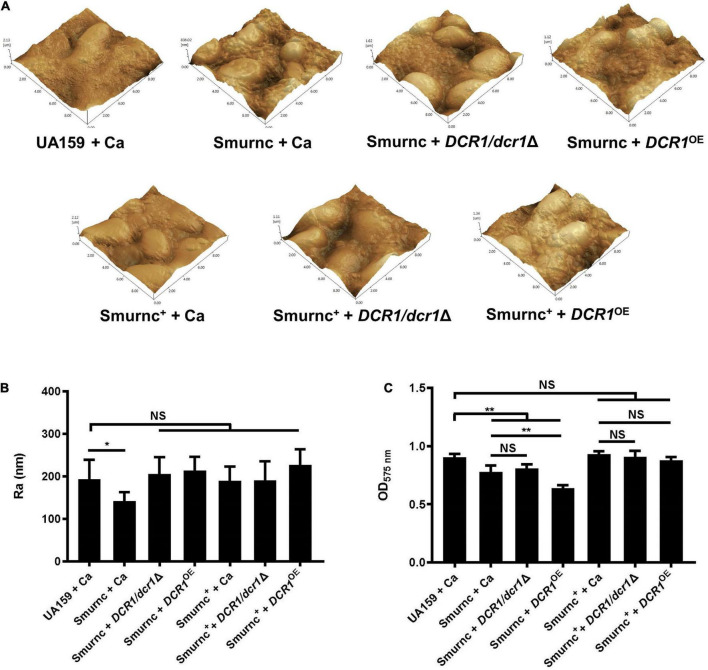
The genes coding RNase III modulated the formation and surface characteristics of cross-kingdom biofilms. **(A,B)** Biofilm morphology and surface roughness (Ra) were tested by AFM. **(C)** Biofilm volume was examined by the CV microtiter assay. Experiments were performed in biological triplicate. *P < 0.05, **P < 0.01.

**FIGURE 5:**
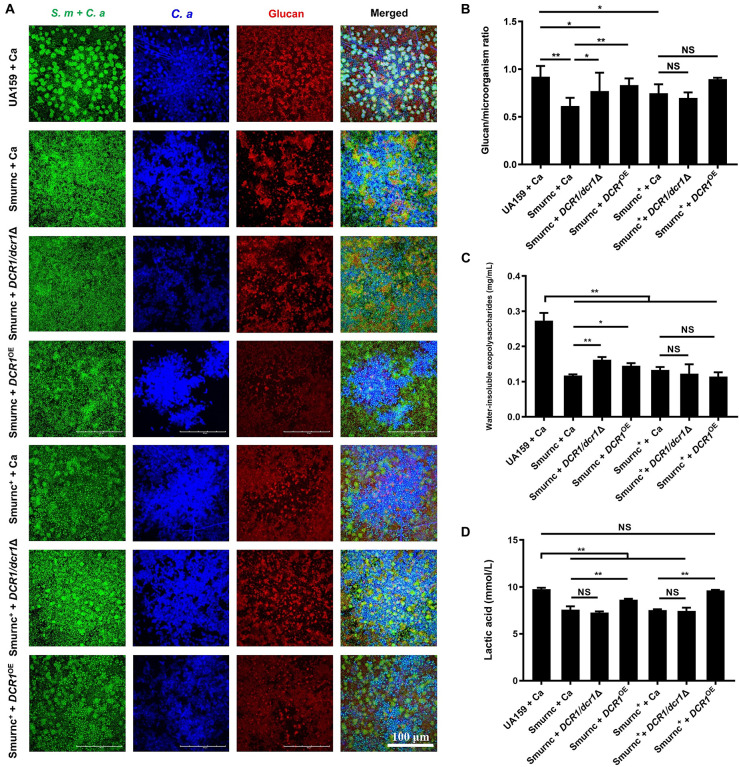
The genes coding RNase III regulated the exopolysaccharides synthesis and lactic acid production of cross-kingdom biofilms. **(A,B)** Exopolysaccharide production and distribution in biofilms were visualized by CLSM. Green, total microbes; blue, *C. albicans*; red, glucan and the ratio of exopolysaccharides to microbes was calculated. **(C)** Water-insoluble exopolysaccharides of biofilms were quantified by the anthrone method. **(D)** Lactic acid production was detected by the Lactic Acid Assay Kit. Experiments were performed in biological triplicate. *P < 0.05, **P < 0.01.

Microorganisms appeared to gather into condensed clusters surrounded by abundant extracellular polymeric substances to form the 3D structure in biofilms of the UA159 + Ca or Smurnc^+^ + Ca group (Figures 2A,D,G). Conversely, the biofilm of Smurnc + Ca had scattered microcolonies with barren extracellular matrix (Figures 2A,D,G); this was in line with the results of quantitative calculation which affirmed that the glucan/microorganisms ratio and total content of water-insoluble EPS became diminished in Smurnc + Ca biofilm when compared to UA159 + Ca or Smurnc^+^ + Ca (Figures 2E,F and Supplementary Figure 3). The fungi among Smurnc + Ca biofilms were predominantly yeast cells, whereas *C. albicans* was present mainly in the hyphal form in the UA159 + Ca or Smurnc^+^ + Ca group (Figures 2A,D). Moreover, the biomass of Smurnc + Ca dual-species biofilms was attenuated significantly, which was accompanied by the conspicuous reduction of biofilm roughness and acid production (Figures 2B,C,H). To further explore the combined effect of *rnc* and *DCR1* on the cariogenic characteristics of cross-kingdom biofilms, *S. mutans rnc* mutant strains were co-cultured with *C. albicans DCR1* mutant strains. There were principally *C. albicans* yeast cells in the biofilms containing Smurnc, *DCR1/dcr1*Δ, or *DCR1*^OE^ (Figures 3, 5A). Notably, when *S. mutans* was defective in *rnc*, the dual-species biofilms were devoid of scaffold structure and had the least and loosest matrix compared to other groups (Figure 3), resulting in planar architecture with exposed microbial cells, reduced biomass, and low surface roughness (Figure 4). AFM detection also revealed that the cells were densely immersed in the flourishing extracellular matrix in the biofilms of Smurnc^+^ + Ca, Smurnc^+^ + *DCR1/dcr1*Δ, and Smurnc^+^ + *DCR1*^OE^, which was similar to the UA159 + Ca group (Figure 4A). Bacteria were sparsely distributed in biofilms composed of Smurnc and *C. albicans* wild-type strain or mutant strains (Figure 5A). Quantitative analysis showed that the Smurnc + Ca biofilm exhibited a markedly lower glucan/microorganism volume ratio, restrained synthesis of water-insoluble EPS, and impaired lactic acid production (Figures 5B–D). Taken together, the *rnc* gene in *S. mutans* has more pronounced regulatory effects on the cariogenic properties of cross-kingdom biofilms than *DCR1*. Consequently, we chose biofilms consisting of UA159 + Ca and Smurnc + *Ca* and Smurnc^+^ + Ca for the following research.

### The *rnc* gene affected virulence factor expression and the pathways of polysaccharide metabolism in *Candida albicans*

**FIGURE 6:**
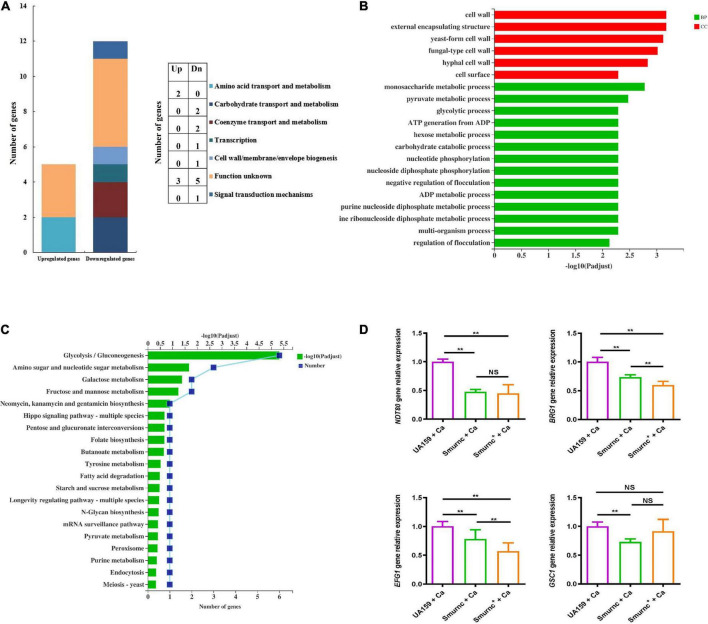
*rnc* gene was associated with the gene expression of virulence factors of *C. albicans*. Transcriptomic analysis of the Smurnc + Ca group compared to the UA159 + Ca group. **(A)** The differently expressed genes were classified by gene annotation based on COG. **(B)** Functional categories and enrichment analysis of differently down-expressed genes based on GO. **(C)** Functional categories and enrichment analysis of differently down-expressed genes based on KEGG. **(D)** The gene transcripts of *C. albicans* in the cross-kingdom biofilms were relatively quantified by RT-qPCR using *ACT1* as a reference gene and interpreted as fold changes based on the UA159 + Ca group. Experiments were performed in biological triplicate. **P < 0.01.

To further investigate the effects of *rnc* on the biological properties of *C. albicans* in the cross-kingdom biofilm, the virulence gene transcription level and pathway alterations were revealed by qRT-PCR, RNA-seq, and bioinformatics. The COG results showed that 12 fungal genes were observably downregulated and 5 fungal genes were upregulated in Smurnc + Ca biofilms compared to UA159 + Ca biofilms (Figure 6A). These genes are related mainly to carbohydrate bioprocess, cell wall biogenesis, signal transduction, and transcription (Figure 6A). GO demonstrated that the majority of downregulated genes were involved in yeast-hyphae transformation, monosaccharide metabolic processes, and pyruvate metabolism (Figure 6B). Through the KEGG pathway analysis, *C. albicans* showed decreased glycolysis/gluconeogenesis, galactose metabolism, and fructose and mannose metabolism (Figure 6C), suggesting that the *C. albicans* pathways associated with carbohydrate metabolism and pyruvate metabolism were suppressed in biofilms of Smurnc + Ca compared to the UA159 + Ca group (Figures 6B,C). Transcriptome data were further confirmed by qRT-PCR which illustrated that the *rnc* mutant strains significantly downregulated the expression of *C. albicans* genes relevant to biofilm formation (*BRG1*, *NDT80*, and *EFG1*) compared with UA159 (Figure 6D). The transcriptional level of the *C. albicans* glucan synthesis gene *GSC1* in Smurnc + *C.a* biofilm was depressed compared to that in UA159 + Ca or Smurnc^+^ + Ca biofilms (Figure 6D).

## Discussion

Biofilms are dynamic and organized communities in which microbial cells are packed with and embraced by EPS to form a 3D structure to resist environmental interference (Lobo et al., 2019; Zhang et al., 2022). Microorganisms carry out complex life activities in biofilms; therefore, expounding the interactions between microorganisms in dual-species biofilms is paramount to developing management strategies for dental caries (Flemming et al., 2016). Among the dental plaque of ECC, *S. mutans* was always detected with *C. albicans* that can also produce acids and survive in an acidic environment, implying the important role of *C. albicans* in biofilm-induced caries (Yang et al., 2012). In previous studies, we found that the *rnc* gene was able to promote the synthesis and configuration of EPS, thus amplifying the cariogenicity of *S. mutans* (Lei et al., 2019, 2020; Lu et al., 2021). Here, our study provided new insights that the *rnc* gene of *S. mutans* and the *DCR1* gene of *C. albicans* modulated extracellular polysaccharide synthesis, thus contributing to the cross-kingdom biofilm formation of *S. mutans* and *C. albicans*.

For the first time, we investigated the effects of the essential gene *DCR1* on the biological characteristics of cross-kingdom biofilms. When the *DCR1* expression level was changed, the arrangement of cell clusters in the biofilm was loose, and the 3D structure formed by the cells and matrix was not obvious (Figures 1A,D). The transformation of cells from yeast to hyphal type is an important virulence factor of *C. albicans* (Carlisle et al., 2009; Sapaar et al., 2014). *C. albicans* hyphae can express a variety of specific virulence factors such as adhesins and antioxidant defense proteins, which participate in the establishment of biofilm structure and immune escape (Greig et al., 2015; Noble et al., 2017). *C. albicans* was present mostly in the yeast form in biofilms containing *DCR1/dcr1*Δ or *DCR1*^OE^ (Figures 1A,D), congruent with the downregulation of mycelium-related virulence factor genes in *DCR1*-mutant strains (Supplementary Figure 2). These results suggest that abnormalities in *DCR1* could change the spatial structure of cross-kingdom biofilms. The roughness of the biofilm surface is conducive to providing a larger area for microbial adhesion and producing surfaces with low shear stress, which can protect the biofilm from elimination (Shen et al., 2015). Nevertheless, we found that the regulatory effect of *DCR1* on biofilm roughness was limited (Figures 1G,H).

Extracellular polysaccharides are the main component as well as the vital cariogenic virulence factor of dental plaque biofilms (Koo et al., 2013). *DCR1* regulated the ability of glucan metabolism of microbes but could not affect the biomass and water-insoluble EPS content of biofilms (Figures 1B,E,F). In dental plaque, the microbiome can ferment carbohydrates to produce lactic acid, reduce the pH of the biofilm microenvironment, and finally cause demineralization of tooth hard tissue (Valm, 2019; Gabe et al., 2020). From this point of view, *DCR1* overexpression presumably facilitates ECC formation by enhancing acid production (Figures 1C, 5D). We conjectured that the gene expression associated with lactic acid production of the *DCR1*^OE^ mutant may be regulated by siRNAs at the post-transcriptional level (Bernstein et al., 2012). Further studies are still needed to investigate the regulatory effects and underlying mechanisms of *DCR1* on acid production of the cross-kingdom biofilm.

Although low- or over-expression of *DCR1* could downregulate virulence genes related to biofilm formation of *C. albicans*, the water-insoluble EPS content and biomass of cross-kingdom biofilms of *S. mutans* and *C. albicans* did not change when *DCR1* was expressed abnormally, implying that there are likely complex interactions between these two strains to seek advantages and avoid disadvantages, thus forming a micro-ecological environment that is more beneficial to resist external adverse factors (Arnold, 2022). In contrast, *S. mutans* was supposed to play a more pivotal role in regulating the formation of dual-species biofilms.

We further explored the role of *rnc* in the cariogenic characteristics of cross-kingdom biofilms. Biofilms with Smurnc lacked an obvious scaffold structure and had less matrix wrapped around the microbes, revealing that *rnc* assisted in the formation of the extracellular matrix of dual-species biofilms and led to changes in their morphological structure. The EPS layer in biofilms was a manifestation of the defective biofilm, which was devoid of stereoscopic architecture. Deletion of the *rnc* gene inhibited the EPS metabolism instead of completely eliminating EPS synthesis in *S. mutans*, consistent with the quantitative data detected by the anthrone assay and the visualized images obtained by CLSM. Therefore, *rnc*-deficient *S. mutans* has defective biofilms but still with an EPS layer compared to the UA159 + Ca group (Figure 3). When compared to the *DCR1* gene, deficiency of *rnc* could conspicuously restrain extracellular polysaccharide synthesis, decrease surface roughness, and cause attenuated acid production, which diminished the construction of cariogenic dual-species biofilms. Meanwhile, changes induced by the lack of *rnc* possibly impacted *C. albicans* virulence by affecting its biological behavior, such as biofilm formation, which may ultimately repress the cross-kingdom biofilm.

*Ndt80*, *EFG1*, and *BRG1* are important transcription factors involved in the formation of *C. albicans* biofilms. After the knockout of these genes, *C. albicans* cannot display normal biofilms *in vivo* or *in vitro* (Nobile et al., 2012). β-1,3 glucan is one of the three major components of the *C. albicans* extracellular matrix, and its synthesis pathway involves mainly the *GSC1* gene (glucan synthase catalytic subunit 1), which amplifies biofilm formation and drug resistance by increasing the extracellular matrix (Mitchell et al., 2015). Based on the phenotype and gene expression results, we speculated that *rnc* expedited the formation of cross-kingdom biofilms through EPS anabolism and catabolism, also propitious to the growth and the upregulated expression of the virulence genes of *C. albicans*. Related biological activities of *C. albicans*, such as hyphal transformation, glucan synthesis, and biofilm development, could further improve the structure of cross-kingdom biofilms and give full play to biofilm cariogenic properties such as acid production. In this study, we elucidated that *S. mutans* may play a more prominent role in regulating the formation of dual-species biofilms. Therefore, although the virulence- or biofilm-associated genes such as *BRG1* and *EFG1* of *C. albicans* were significantly lower in Smurnc^+^ + Ca than in Smurnc + Ca, Smurnc + Ca might have defective biofilms because of the impaired EPS synthesis and biofilm establishment induced by the absence of *rnc* (Figure 6D). Moreover, the cross-kingdom biofilm of *S. mutans* and *C. albicans* may be modulated by *C. albicans* virulence genes in addition to *BRG1* and *EFG1*, which need to be further explored.

Transcriptomics helped us clarify the specific molecular mechanism and main pathway by which the *rnc* gene affects *C. albicans* (Motaung et al., 2017). It is possible that the phenotype of different morphological transformations of *C. albicans* in the Smurnc + Ca group was attributed to the downregulation of cell wall-related genes (Figure 6B). The downregulated genes in glycometabolism pathways suggested that the deletion of *rnc* in *S. mutans* possibly impacted the polysaccharide synthesis of *C. albicans* in the cross-kingdom biofilms, leading to the disruption of biofilm formation. Pyruvate metabolism is an important way to generate lactic acid. In the Smurnc + Ca group, the significantly decreased lactic acid production might be associated with the weak acid production capacity of *C. albicans* caused by the downregulation of genes relevant to the pyruvate metabolism pathway.

In conclusion, both RNase III coding genes of *S. mutans* and *C. albicans* can regulate the biological characteristics of the cross-kingdom biofilm. The *rnc* gene of *S. mutans* was able to crucially promote the formation of cross-kingdom biofilms with cariogenic characteristics by affecting the synthesis and metabolism of EPS. At the same time, changes in the dual-species environment were conducive to hyphal transformation, biofilm formation, polysaccharide metabolism, and pyruvate metabolism in *C. albicans*. The *DCR1* gene of *C. albicans* mainly played a role in improving the biofilm structure and affecting the development of the acidic microecological environment. This research may provide an avenue for exploring new targets for the effective prevention and treatment of ECC.

## Data availability statement

The data presented in the study are deposited in the NCBI repository under BioProject accession number PRJNA854798 (https://www.ncbi.nlm.nih.gov/sra/PRJNA854798).

## Author contributions

TH, YY, and XW designed the experiments and revised and finalized the manuscript. YL, LL, and YD performed the experiments, analyzed the data, and wrote and finalized the manuscript. HZ and MX contributed to data collection and analysis. All authors contributed to the article and approved the submitted version.
