# Supplementary material for: RNase III coding genes modulate the cross-kingdom biofilm of Streptococcus mutans and Candida albicans
Source: Front Microbiol. 2022 Sep 30;13:957879. doi: 10.3389/fmicb.2022.957879 (PMC9563999; doi:10.3389/fmicb.2022.957879)
Supplement: Supplementary file 1 [file Data_Sheet_1.docx]

Supplementary Material

**RNase III coding genes modulate the cross-kingdom biofilm of *Streptococcus mutans* and *Candida albicans***

Yangyu Lu^1,2†^, Lei Lei^1†^, Yalan Deng^1†^, Hongyu Zhang^1^, Mengying Xia^1^, Xi Wei^2^, Yingming Yang^1*^, Tao Hu^1*^

^1^ State Key Laboratory of Oral Diseases, National Clinical Research Center for Oral Diseases, Department of Preventive Dentistry, West China Hospital of Stomatology, Sichuan University, Chengdu, China

^2^ Department of Operative Dentistry and Endodontics, Hospital of Stomatology, Guanghua School of Stomatology, Sun Yat-sen University, Guangdong Provincial Key Laboratory of Stomatology, Guangzhou, China

^†^These authors have contributed equally to this work

*** Correspondence:**

Tao Hu

1. mail: [hutao@scu.edu.cn](mailto:hutao@scu.edu.cn)

Yingming Yang

E-mail: [ymyang@scu.edu.cn](mailto:leilei@scu.edu.cn)

**TABLE S1. Oligonucleotide primers used in this study**

| **Primers** | **Nucleotide sequences (5’-3’)** | **Size (bp)/Refernce** |
| --- | --- | --- |
| **PCR** | | |
| DCR1-chk-F | ATTGCCGTTGGTCCAGCAAGTAATG | About 1600 bp |
| hisG-chk-R | GTAGATAACTTCGACTTCACGCAGG |  |
| hisG-chk-F  DCR1-chk-R  DCR1-R  RP10-R  **RT-qPCR** | GGAACGCAGAATGTTAGACAACACC  TATGGGTTGACAGTTCCCTATGACG  TTCTGGTGAGTCCTTAGTTCGGAAATC  CTCGTATTCACTTAATCCCACACTC | About 2000 bp  About 4000 bp |
| *ACT1* | FW- GAAGAAGTTGCTGCTTTAG  RV- CGTCGTCACCGGCAAAA | (Bhattacharya et al., 2016) |
| *URA3* | FW- TAATGCTCATGGTGTCACTG  RV- CAAATCCTTCTTCTTGTCCA | (Brand et al., 2004) |
| *NDT80* | FW- GGCGAACTGAGAGTTCCAATTA  RV- GTGGGAACTGTGGACCTTTATC | (Glazier et al., 2017) |
| *BRG1* | FW- GGTCATATAATAGCAGTGCA  RV- ATAGTGTAACCCACATTAGG | (Basso et al., 2017) |
| *EFG1* | FW- TACCAGGTCAACAAGCAGTACCTAT  RV- ACATGGTAGTTGTTACTCGTGGTCT | (Basso et al., 2017) |
| *GSC1* | FW- TCAACAACAACCATATGACATGGA  RV- ACCACCATAACTAAAGTCAGAAAAG | (Roman et al., 2015) |


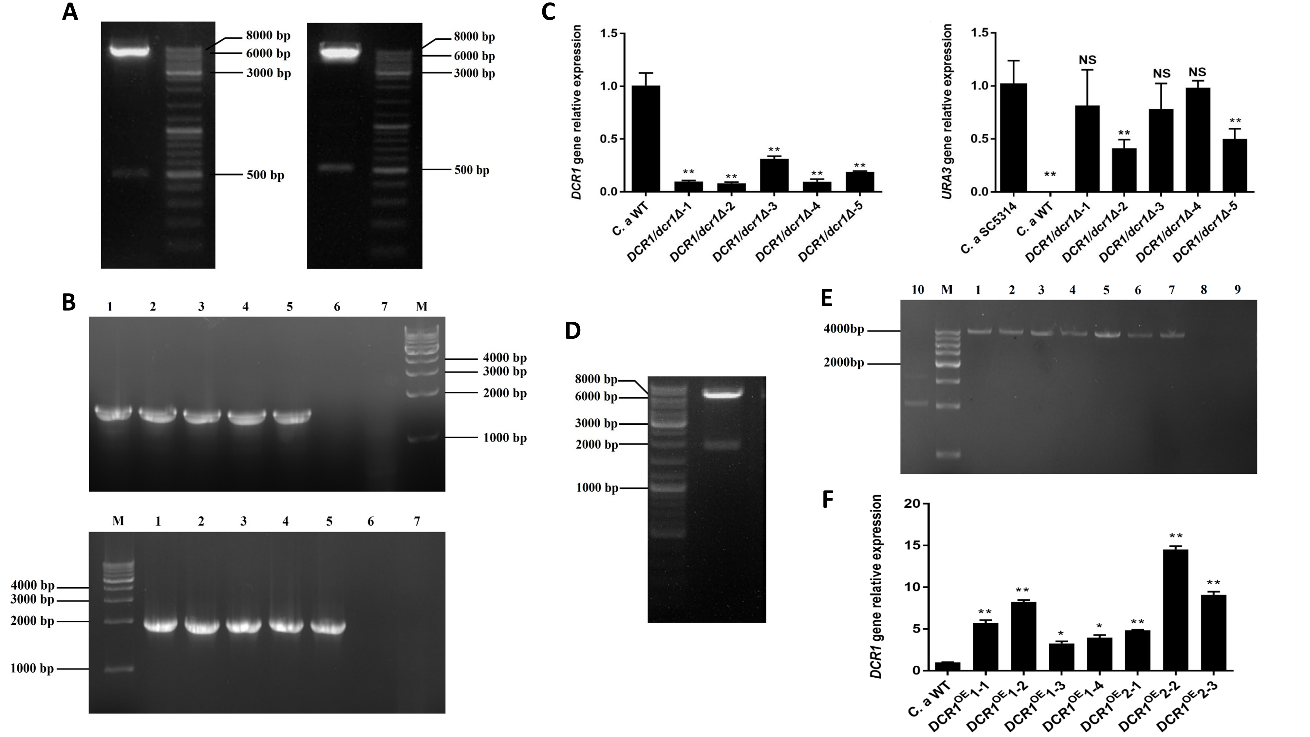


**FIGURE S1 Construction of *C. albicans* *DCR1/dcr1*Δ and *DCR1*^OE^ strains.** (A) Recombinant plasmid pUC-*DCR1*-*URA3* was verified by [gel](javascript:;) [electrophoresis](javascript:;). (B) *DCR1/dcr1*Δ strains were verified via PCR. Lane 1-5: Transformants. Positive clones had specific amplified fragments about 1600-2000 bp; Lane 6: *C. albicans* WT; Lane 7: pUC-*DCR1*-*URA3*; M: Marker. (C) *DCR1/dcr1*Δ strains were verified by RT-qPCR. (D) Recombinant plasmid pCaEXP-*DCR1* was verified by [gel](javascript:;) [electrophoresis](javascript:;). (E) *DCR1*^OE^ strains were verified via PCR. Lane 1-8: Transformants. Positive clones had specific amplified fragments about 4000 bp; Lane 9: *C. albicans* WT; Lane 10: pCaEXP- *DCR1*; M: Marker. (F) *DCR1*^OE^ strains were verified by RT-qPCR. **P*< 0.05, ***P*< 0.01.


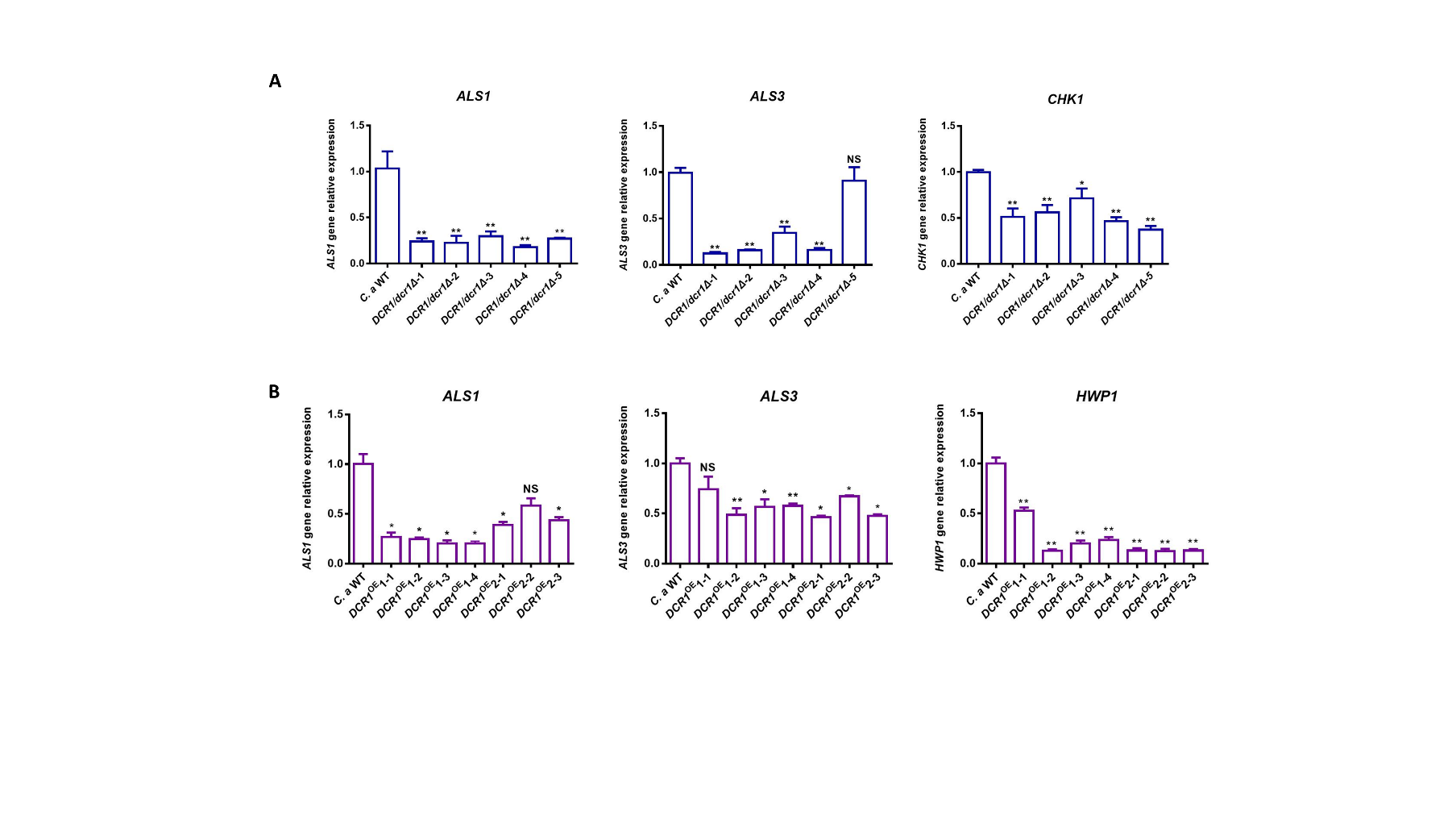


**FIGURE S2 Virulence factors expression of *C. albicans* *DCR1*-mutant strains were measured by RT-qPCR.** (A) Expression level of genes relevant to hyphal transformation (*HWP1*), adherence and biofilm formation (*ALS3* and *CHK1*) in *DCR1/dcr1*Δ planktonic cells. (B) Expression level of *HWP1*, *ALS1* and *ALS3* in *DCR1*^OE^ planktonic cells.

**
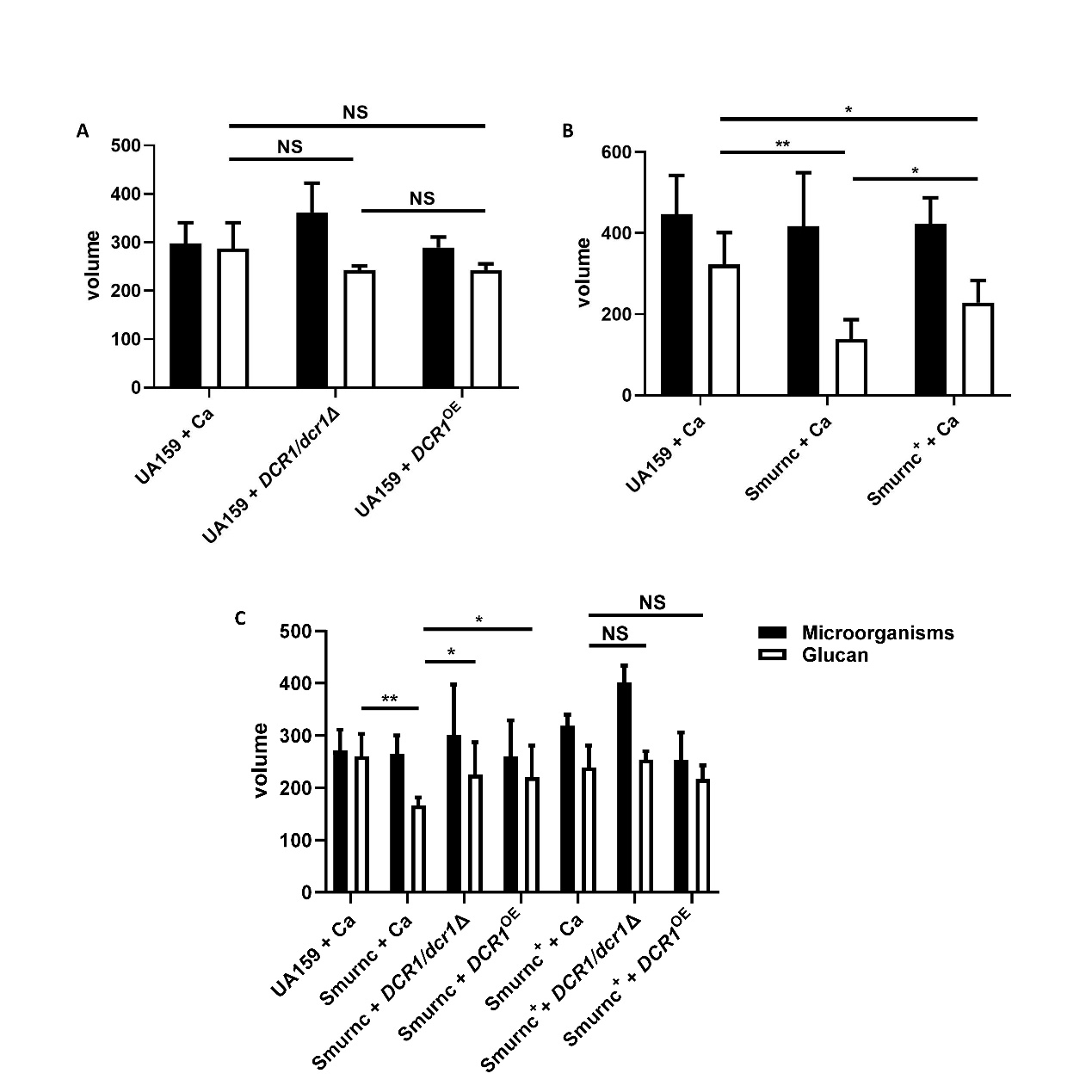
**

**FIGURE S3 Quantitative data of microorganisms and glucan in the cross-kingdom biofilms were detected by confocal laser scanning microscopy.** **P*< 0.05, ***P*< 0.01.

**Reference**

Basso, V., Znaidi, S., Lagage, V., Cabral, V., Schoenherr, F., LeibundGut-Landmann, S., et al. (2017). The two-component response regulator Skn7 belongs to a network of transcription factors regulating morphogenesis in Candida albicans and independently limits morphogenesis-induced ROS accumulation. *Mol Microbiol* 106(1)**,** 157-182. doi: 10.1111/mmi.13758.

Bhattacharya, S., Sobel, J.D., and White, T.C. (2016). A Combination Fluorescence Assay Demonstrates Increased Efflux Pump Activity as a Resistance Mechanism in Azole-Resistant Vaginal Candida albicans Isolates. *Antimicrob Agents Chemother* 60(10)**,** 5858-5866. doi: 10.1128/AAC.01252-16.

Brand, A., MacCallum, D.M., Brown, A.J., Gow, N.A., and Odds, F.C. (2004). Ectopic expression of URA3 can influence the virulence phenotypes and proteome of Candida albicans but can be overcome by targeted reintegration of URA3 at the RPS10 locus. *Eukaryot Cell* 3(4)**,** 900-909. doi: 10.1128/EC.3.4.900-909.2004.

Glazier, V.E., Murante, T., Murante, D., Koselny, K., Liu, Y., Kim, D., et al. (2017). Genetic analysis of the Candida albicans biofilm transcription factor network using simple and complex haploinsufficiency. *PLoS Genet* 13(8)**,** e1006948. doi: 10.1371/journal.pgen.1006948.

Li, D.D., Zhao, L.X., Mylonakis, E., Hu, G.H., Zou, Y., Huang, T.K., et al. (2014). In vitro and in vivo activities of pterostilbene against Candida albicans biofilms. *Antimicrob Agents Chemother* 58(4)**,** 2344-2355. doi: 10.1128/AAC.01583-13.

Roman, E., Alonso-Monge, R., Miranda, A., and Pla, J. (2015). The Mkk2 MAPKK Regulates Cell Wall Biogenesis in Cooperation with the Cek1-Pathway in Candida albicans. *PLoS One* 10(7)**,** e0133476. doi: 10.1371/journal.pone.0133476.
